# Salud de la comunidad LGBTQ+ y atención primaria

**DOI:** 10.1016/j.aprim.2022.102377

**Published:** 2022-06-05

**Authors:** Mauro Tamayo Rozas, Camila Herrera Ureta, Marión Stock, Álvaro Besoaín-Saldaña

**Affiliations:** a Municipalidad de Cerro Navia, Cerro Navia, Región Metropolitana, Chile; b Departamento de Kinesiología, Facultad de Medicina, Universidad de Chile, Independencia, Región Metropolitana, Chile; c Núcleo Desarrollo Inclusivo, Universidad de Chile, Independencia, Región Metropolitana, Chile

*Señor Editor:*

El término LGBTIQA+ incorpora a las orientaciones sexuales e identidades de género como lesbiana, gay, bisexual, transgénero, intersexual, queer y otras identidades u orientaciones de género. Se ha identificado que esta comunidad tiene necesidades de salud que no suelen ser bien comprendidas en los servicios de salud^1^, ^2^. Las necesidades insatisfechas de esta comunidad suelen ser descritas como desigualdades en salud y reconocidas como un problema de equidad del sistema^3^ expresado en falta de acceso y calidad por parte de la red asistencial, necesidades específicas como terapia vocal, pesquisa en salud mental y control de terapia hormonal^1^, ^2^, ^3^.

El sistema de salud chileno está conformado por un sistema mixto (público y privado) de financiamiento, aseguramiento y provisión de servicios. La rectoría es entregada por el Ministerio de Salud. Dentro del subsistema público, la Atención Primaria en Salud (APS) es esencialmente administrada por los municipios^4^, instituciones con la responsabilidad de desarrollar además funciones relacionadas con servicios sociales y culturales, entre otras. Si bien las redes de servicios se articulan con la APS para asegurar la continuidad asistencial, esta se ve limitada por la falta de comunicación y coherencia entre niveles. En este marco, las municipalidades generan estrategias sobre grupos poblacionales excluidos para asegurar integralidad y continuidad. No obstante, su acción sobre promover espacios seguros, comunitarios y respetuosos de las comunidades LGBTIQA+ aún está pendiente y presenta desafíos estructurales. En Chile, solo 56 municipios de 345 cuentan con oficinas de diversidad o disidencias sexuales, según RedDiversa^a^.

La Municipalidad de Cerro Navia de Chile, decidió incorporar procesos intersectoriales de apoyo, acompañamiento e investigación en salud de la comunidad LGBTIQA+^b^. Durante diciembre de 2021, la Oficina de las Diversidades y Disidencias de esta institución realizó una investigación junto a miembros de la comunidad LGBTIQA+ y estudiantes de la Facultad de Medicina de la Universidad de Chile para analizar el efecto del uso de Binder^5^, una camiseta compresora que se usa para reducir la notoriedad del pecho, además de la importancia de informar sobre este tema a las y los funcionarios de salud y evitar situaciones de mala atención o discriminación^6^.

Se desarrolló un estudio cuantitativo, observacional y de corte transversal con un muestreo por conveniencia de 72 personas mayores de 18 años. Del total de quienes respondieron la encuesta, el 63,9% dijeron haber usado Binder. Más del 60% se encontraban entre los 18-24 años. Solo el 10,9% de los usuarios de Binder consultaron con algún profesional de la salud. Las principales razones para no consultar fueron no creer que los profesionales manejan el tema (67,4%), no haberlo encontrado necesario (48,8%), no considerarlo un espacio seguro (37,2%) y haber sentido miedo o haberse sentido juzgado (25,6%). En cuanto a efectos físicos en salud, los efectos adversos más frecuentes fueron calor excesivo (80,4%), dificultad para respirar (67,4%) y dolor de espalda (60,9%). Sobre las implicancias en salud mental, el 91,3% reportaron aumento de confianza en espacios públicos y el 86,9% aumentaron su autoestima. A su vez, el 39,1% reportaron disminución de trastornos ansiosos, el 36,9% de trastornos depresivos y el 76% de disforia de género.

En ese marco, se han desarrollado procesos de capacitación en equipos de APS sobre necesidades de la comunidad LGBTIQA+ para articular una respuesta pertinente, respetuosa e inclusiva. Como resultado de la investigación se desarrolló una guía sobre el uso adecuado del Binder que implica asistir al centro de atención primaria más cercano. Es urgente incentivar espacios institucionales que coordinen de forma intersectorial acciones que promuevan el respeto irrestricto a los derechos humanos de la comunidad LGBTIQA+.

## Financiación

Esta experiencia fue desarrollada por la Municipalidad de Cerro Navia, Región Metropolitana, Chile.

## Autoría

La experiencia descrita es parte del trabajo del autor MT y las autoras CH y MS. El proceso fue sistematizado por AB y trabajado en el marco de un proceso colaborativo entre la Universidad y la Municipalidad.

## Conflicto de intereses

Los autores declaran no tener ningún conflicto de intereses.

## Footnotes

a Red Nacional de Municipalidades por la Diversidad, Inclusión y No Discriminación. Información disponible en: https://www.reddiversa.cl/estudios Consultas a: contacto@reddiversa.cl

b Municipalidad de Cerro Navia, Oficina de las Diversidades y Disidencias Sexuales (2022). Guía de cuidados para usuarios de Binder: https://www.cerronavia.cl/guia-de-cuidados-para-uso-de-binder/
